# Types of leisure-time physical activity participation in childhood and adolescence, and physical activity behaviours and health outcomes in adulthood: a systematic review

**DOI:** 10.1186/s12889-024-19050-3

**Published:** 2024-07-04

**Authors:** Kelcie Miller, Claire Morley, Brooklyn J. Fraser, Seana L. Gall, Verity Cleland

**Affiliations:** 1 Menzies Institute for Medical Research, University of Tasmania, 17 Liverpool St, Hobart, 7000 Australia; 2 School of Clinical Sciences, Monash University, Melbourne, Australia; 3 School of Exercise and Nutrition Sciences, Deakin University, Geelong, Australia; 4 Private Bag 23, Hobart, 7001 Australia

**Keywords:** Sports, Adolescence, Physical activity, Longitudinal, Preventive Health, Cardiovascular Risk, Mental Health

## Abstract

**Background:**

Youth leisure-time physical activity participation benefits physical activity habits and health outcomes later in life. However, it is unknown if certain types of leisure-time physical activity contribute to these benefits in different ways; this knowledge could enhance public health efforts. This systematic review aimed to synthesise evidence of the longitudinal associations between childhood and adolescent leisure-time physical activity on adulthood physical activity behaviours and health outcomes.

**Methods:**

A systematic search of the literature was conducted across five databases from inception to July 2022. English, peer-reviewed observational studies with a minimum of two timepoints of data collection were eligible for inclusion. We included studies that investigated the association between participation in leisure-time physical activity types in children and adolescents (i.e., 5–18 years), and physical activity, mental health, or cardiovascular outcomes in adulthood (i.e., ≥ 18 years).

**Results:**

Fourteen studies were included in the review, totalling 34,388 observations across five countries. Running in adolescence was associated with increased adulthood physical activity in both sexes, while sports involvement was associated with an increase in physical activity in males only. Adolescent team sports participation was associated with reduced odds of early adulthood depression, with varying findings for anxiety disorders. There was preliminary evidence of minimum threshold requirements for participation in certain activities before associations with future physical activity or health outcome benefits were observed.

**Conclusions:**

Preliminary findings suggest that the lifelong behavioural and health benefits of adolescent participation in leisure-time physical activity appear to be related to the type of activity undertaken, with potential differences between sexes. With the rarity of longitudinal studies spanning from childhood into adulthood, these findings provide important insights for public health strategies to optimise lifelong health and physical activity participation.

**Prospero registration:**

CRD42022347792.

**Supplementary Information:**

The online version contains supplementary material available at 10.1186/s12889-024-19050-3.

## Introduction

Undertaking regular physical activity (PA) across the lifespan is vital for ensuring optimal physical and mental health. PA participation throughout life is associated with a reduced risk of mortality and chronic health conditions, including breast and colon cancers, type 2 diabetes, cardiovascular disease, osteoporosis, dementia, and depression [1–3]. Physical inactivity accounts for approximately 6% of mortality worldwide and is the fourth leading risk factor for developing non-communicable diseases [4]. Furthermore, childhood PA participation benefits PA behaviours [5], mental health [6, 7], and cardiovascular disease outcomes [8] later in life. Subsequently, encouraging lifelong PA participation is of public health interest. Despite this, 27.5% of adults globally remain insufficiently active, with women less active than men [3, 9]. To optimise public health, a better understanding of PA participation behaviours and their relationship with health outcomes is needed.

PA can be classified into several domains: leisure-time, transport-related, domestic, and occupational or school-based PA [5]. Leisure-time physical activity (LTPA) is a major and modifiable component of an individual’s PA, referring to physical activities that are undertaken voluntarily, and in an individual’s discretionary time [10].This may include sports participation, walking, strength-training, or other recreational activities. LTPA can be further categorised by the mode (e.g., organised, non-competitive, non-organised), setting (e.g., school, club, neighbourhood), and type of activity (e.g., tennis, walking) [11]. Compulsory school-based physical activity such as physical education is generally not considered a component of LTPA, as this occurs outside of an individual’s freely disposable time. Previous LTPA-focussed research has mostly been quantified by time, frequency, and/or intensity (e.g., light intensity, moderate-vigorous intensity), with little investigation into the impact of the specific activity types of LTPA being performed [12–15].

The World Health Organisation Physical Activity and Sedentary Behaviour Guidelines Development Group [14] identified understanding the health effects of different PA types as one of four major evidence gaps in current knowledge. Therefore, summarising the current state of knowledge of the longitudinal health and behavioural effects of specific LTPA types could help inform public health policy and direct future research. Targeting life stages in which PA participation is volatile is key to promoting consistent PA engagement. As adolescence and early-adulthood are significant timepoints for shifts in LTPA participation, it is important that these life stages are considered [16, 17]. As such, understanding which types of LTPA participation in early life may best promote positive lifelong PA behaviours and outcomes would be beneficial. Such knowledge may assist in reducing the global burden of major diseases, including cardiovascular disease and mental health disorders, and further, achieving PA targets for improved population health.

Cardiovascular health, mental health, and physical inactivity each impose a major public health burden globally. Insufficient physical activity is estimated to be responsible for 20–30% of premature deaths [3], making physical activity promotion a public health priority [18]. With cardiovascular disease the leading cause of mortality worldwide, responsible for approximately 17.9 million deaths each year [19], and the prevalence of mental health disorders at 16% of the world’s population [20, 21], understanding mechanisms and interventions that prevent or improve these conditions is imperative to bettering public health. While early PA participation is known to be positively associated with adulthood PA, mental health, and cardiovascular health, the specific activity types that most optimally benefit health outcomes remain unclear.

Previous systematic reviews have highlighted the influence of activity structure and type on psychosocial outcomes in children [22] and cardiovascular health in adults [23]. Another review identified a largely positive relationship between overall childhood sport participation and adulthood PA [24]. However, the longitudinal associations between participation in specific LTPA types during childhood and adolescence and adult health and PA behaviours remains unknown. Therefore, this systematic review aims to collate previous findings on the associations between participation in specific types of LTPA in childhood and adolescence, and PA behaviours and mental and cardiovascular health-related outcomes in adulthood.

## Methods

### Protocol and registration

The systematic review protocol was registered with the International Prospective Register of Systematic Reviews (PROSPERO; registration number CRD42022347792). The review has been reported in line with the Preferred Reporting Items for Systematic Reviews and Meta-Analyses (PRISMA) statement [25]. A completed PRISMA Checklist and PRISMA for Abstracts Checklist can be seen in Additional file 1.

### Selection criteria

Studies were eligible for inclusion if they met the following criteria:

(i) Full-text original peer-reviewed journal article published in English;
(ii) observational study design on humans with at least two timepoints of data collection;
(iii) cohort from the general population,
(iv) measurement of at least one specific type of LTPA (e.g., tennis, walking, strength-training), or grouping of specific types of LTPA with similar attributes (e.g., team sports, ball sports), in childhood or adolescence (aged 5–18 years);
(v) quantitative assessment of at least one mental health outcome (e.g., depression or anxiety symptoms/diagnosis), cardiovascular health outcome (e.g., BMI, waist circumference, cholesterol, cardiovascular events/disease), or PA outcome in adulthood (aged 18 + years).

Studies were excluded based on the presence of the following criteria:

(i) Focussed on targeted populations (i.e., populations with known disease or special interest groups);
(ii) considered setting or mode of activity instead of type (e.g., non-organised vs. organised sport, school vs. non-school setting).

No date restrictions were imposed on the search.

### Information sources

Studies for inclusion in the review were identified by a single researcher searching electronic databases from inception to 27 July 2022. The databases searched included Medline (via OVID), SPORTDiscus (via EBSCOhost), Embase (via OVID), Scopus, and Web of Science.

### Search strategy

The electronic database search strategy was designed with input from an academic librarian experienced in conducting systematic reviews. Derived based on input from several preliminary searches, the search strategy included keywords and Boolean operators defining children and adolescents (childhood, adolescen*, youth, school, teen), physical activity (physical activit*, sport*, exercise*), adulthood (adult*, midlife), and type (type, subtype, participation). A full search strategy, by database, can be found in Additional file 2. No filters were used during the searches.

### Selection process

Records from each database search were imported into EndNote 20 where duplicates were removed. Remaining records were then uploaded to Covidence systematic review management software [26] for a final duplication detection test. Two researchers (K.M and C.M) independently screened the titles and abstracts against the review eligibility criteria, with consensus between the two reviewers required for further screening. Full-text screening was also performed independently by the same two researchers (K.M and C.M), with consensus required for final inclusion. Any discrepancies between the reviewers during this process were resolved by communication or a third reviewer (V.C), if required.

### Data collection

Descriptive data and results from included studies were extracted using a study-specific form created and completed in Covidence software [26]. Data extraction was completed independently by the two researchers (K.M and C.M) that conducted the screening process. Extracted data was compared in Covidence software [26], with any discrepancies resolved by review and discussion between the two researchers (K.M and C.M). Final data were then exported from Covidence to Excel.

### Data items

Data extracted from each eligible study included authors, publication year, country, participant demographics (e.g., age at baseline and follow-up, sex/gender, race), length of follow-up, cohort/study name, sample size, response rate, study design, exposures (i.e., types of PA), data analyses (including adjustments), and outcome data (i.e., cardiovascular health, mental health, and PA measures).

### Risk of bias in individual studies

The quality and risk of bias of included studies was assessed via a modified version of the Newcastle – Ottawa Scale (NOS) for cohort studies [27] (see Additional file 3). Using this scale, the risk of bias of individual studies was assessed across three categories: participant selection and classification, comparability of participants, and outcome assessment. Two researchers (K.M and C.M) independently assessed the risk of bias (quality) of the included records and if necessary, discrepancies between reviewers were resolved by communication or a third reviewer (V.C) prior to reaching consensus. Studies were then categorised as good, fair, or poor quality based on the collated assessment of the three NOS categories. Further details of the scoring system and individual risk of bias results are reported in Additional file 3.

### Data synthesis

Data from the included studies were collated by narrative synthesis due to the methodological and analytical differences present between studies.

## Results

### Study selection

**Fig. 1:**
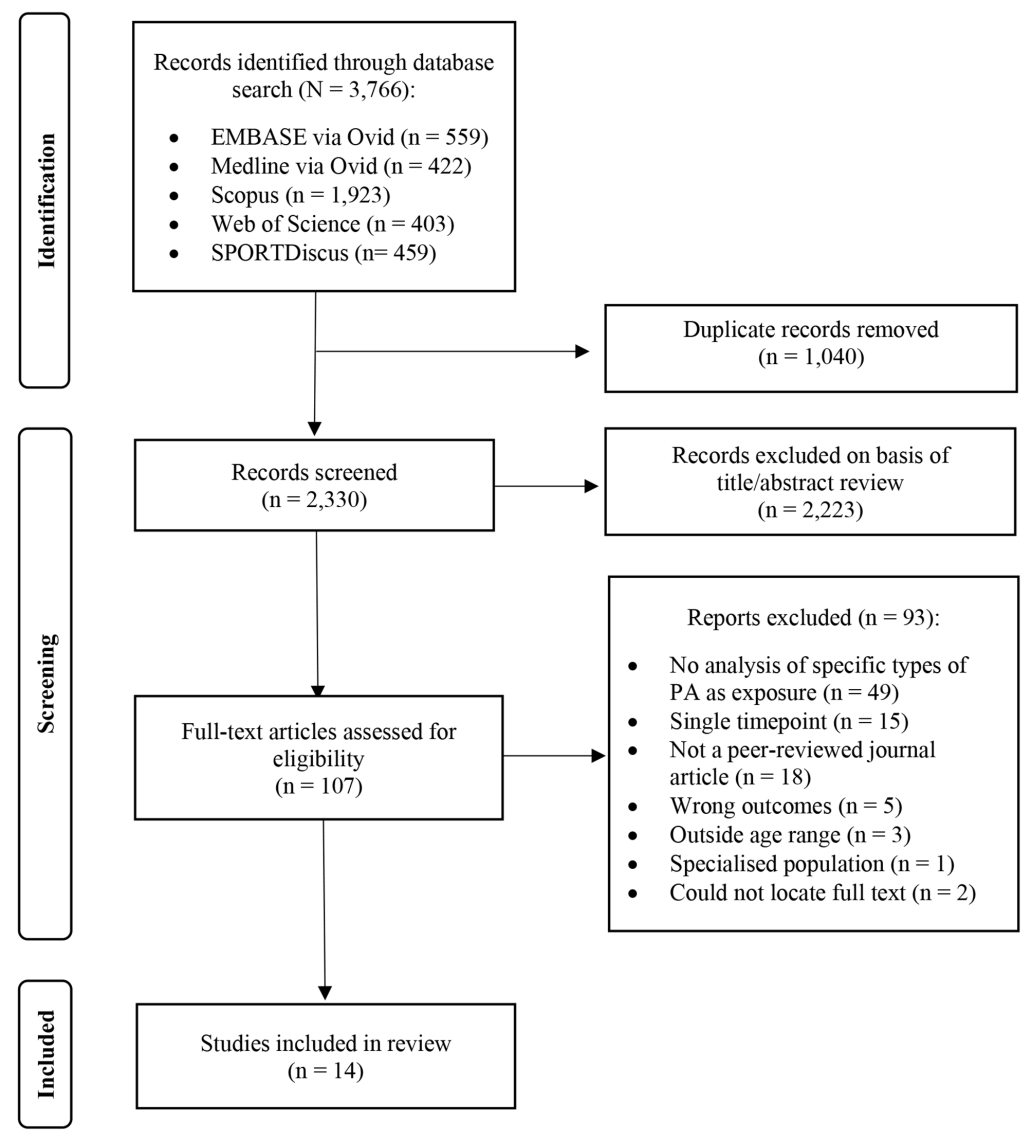
PRISMA flowchart outlining the selection of studies

A total of 3,766 records were identified, and after removing duplicates, 2,330 unique records were retained for screening (Fig. 1). Following title and abstract screening, 107 articles were retained for full-text screening. After the final stage, fourteen articles were deemed eligible for inclusion.

### Study characteristics

Of the fourteen studies included, five investigated early to mid-adulthood PA behaviours as an outcome [28–32], two explored cardiovascular disease risk factors as an outcome [33, 34], and six focussed on mental health outcomes [35–40] (Table 1). One study analysed both mental and cardiovascular health-related outcomes [41]. Included studies were published between 2003 and 2021. The studies utilised data from six cohorts, totalling 34,388 observations, and spanning across five countries: Canada, United States, Sweden, Norway, and Finland. Baseline ages ranged from 12 to 18 years, and outcome measurements were taken between 18 and 53 years, with an average follow-up of fourteen years.

**Table 1:** Summary of included studies, by outcome

Study ID (Author, Year)	Country	Cohort	*N*	Sex, % Male	Age, Baseline (Years)	Age, Follow-up (Years)	Activity Types, Exposure	Outcome Description	Study Quality
**Physical Activity**									
Belanger, 2016 [29]	Canada	NDIT	673	46%	12–13	24	Sports (ice hockey, ice skating, football, roller blading/skateboarding, basketball, soccer, boxing and baseball), fitness/dance, running/walking	Weekly estimate of physical activity (MET minutes/week) using IPAQ-SF	Good
Engström, 2008 [28]	Sweden	-	1518	Not reported	15	53	Team ball games, individual ball games, skiing, track and field/jogging/orienteering, swimming, gymnastics/dance/ballet, walking/cycling	Level of exercise (exerciser vs. non-exerciser)	Fair
Kjonniksen, 2008 [30]	Norway	Norwegian Longitudinal Health Behaviour Study	630	38%	13	23	Soccer, water-skiing, alpine skiing, cross-country skiing, judo/karate, power sports, swimming/diving, ball games, cycling, jogging, bodybuilding, cycling, hard work, walking/hiking	Frequency of moderate-vigorous physical activity (times per week)	Fair
Mäkelä, 2017 [31]	Finland	FinnTwin16 project	3205	43%	17	34	Endurance, power, games, other	Leisure-time MET	Fair
Tammelin, 2003 [32]	Finland	Northern Finland Birth Cohort 1966	7794	47%	14	31	Ice hockey, soccer, volleyball, basketball, other ball games, cross-country skiing, running, swimming, cycling, walking, orienteering, skating, track and field, gymnastics, downhill skiing, riding, dancing, combat sports, strength-training	Frequency of participation in light and brisk physical activity (very active, active, moderately active, inactive)	Good
**Mental Health**									
Ashdown-Franks, 2017 [40]	Canada	NDIT	781	45%	12–13	20	Team sports, individual sports	Panic disorder, generalised anxiety disorder, social phobia, agoraphobia	Fair
Bohr, 2019 [36]	United States	Add Health	10,951	46%	12–18	24–32	Contact sports, non-contact sports	CES-D score, depression diagnosis, suicide ideation, suicide attempts (past 12 months)	Fair
Brunet, 2013 [37]	Canada	NDIT	816	47%	12–13	20	Organised team sports	Frequency of MVPA (days per week) using IPAQ, depressive symptoms (past 14 days)	Good
Deshpande, 2020 [41]	United States	Add Health	2197	100%	12–18	24–32	American football	CES-D score; depression diagnosis, anxiety, post-traumatic stress disorder	Good
Murray, 2021 [38]	Canada	NDIT	733	46%	12–13	20	Team sports	Depressive symptoms, stress, coping, anxiety symptoms (past 14 days)	Good
Sabiston, 2016 [39]	Canada	NDIT	860	46%	12–13	20	Team sports	Depressive symptoms	Good
Viau, 2015 [35]	Canada	-	254	38%	14–17	21	Team sports, individual sports	Depressive symptoms	Fair
**Cardiovascular Health**									
Belanger, 2018 [33]	Canada	NDIT	631	46.0%	12–13	24	Sports (ice hockey, ice skating, football, roller blading/skateboarding, basketball, soccer, boxing and baseball), fitness/dance, running/walking	Height, weight, waist circumference, triceps and subscapular skinfold thickness	Good
Deshpande, 2020 [41]	United States	Add Health	2197	100%	12–18	24–32	American football	Hypertension, high cholesterol/triglycerides, heart disease	Good
Menschik, 2008 [34]	United States	Add Health	3345	49.1%	13–18	18–26	Skating/cycling, sports, fitness/running/dance	BMI	Fair

**Add Health** = National Longitudinal Study of Adolescent Health; **BMI** = body mass index; **CES-D** = Center for Epidemiological Studies-Depression; **IPAQ** = International Physical Activity Questionnaire; **IPAQ-SF** = International Physical Activity Questionnaire - Short Form; **MET =** Metabolic Equivalent of Task; **NDIT =** Nicotine Dependence in Teens

### Risk of bias within studies

Seven studies were rated as good quality using the NOS, with the remaining seven studies rated as fair (Table 1 and Additional file 3). A consistent shortcoming was in the ascertainment of exposure and outcome measurements, with nearly all studies using self-report measures, excluding one [33].

### Synthesis of results

#### Physical activity

Five studies considered adulthood PA outcomes, comprising 13,820 observations [28–32]. The cohorts included were located across five countries: Canada, United States, Sweden, Norway, and Finland. All PA exposures and outcomes were measured by self-report questionnaires, with participants aged between 13 and 17 years at baseline and 23 to 53 years at follow-up.

Kjønniksen and Torsheim, et al. [30] found that, for most activities, participation in a specific activity in adolescence was associated with higher odds of engaging in that same activity ten years later. Adolescent sports participation (i.e., ice hockey, ice skating, football, roller blading/skateboarding, basketball, soccer, boxing and baseball) was associated with higher total PA in early adulthood in one study [29]. When stratifying by sex, soccer [30], volleyball and ice hockey [32] showed associations with higher total early adulthood PA in males. No significant associations were observed for females participating in sports.

Endurance-based activities including running, track and field, and orienteering were positively associated with total adulthood PA [28–30, 32].One study observed a positive effect of walking/hiking on future PA [30], however other studies found no association [28, 32]. Two studies found no association of adolescent swimming participation on early to mid-adulthood PA [28, 32], however another observed a negative association in females [30]. A positive association of adolescent cycling participation on early to mid-adulthood PA was observed in females [32], but not males [28, 32]. Adolescent participation in gymnastic and dance activities was associated with higher mid-adult PA in some studies [28, 32], yet another reported no association [29].

Females engaging in both endurance and power activities during adolescence had higher early adult PA levels compared to females engaging in other combinations of activities [31]. Combat sport participation was positively associated with early adulthood PA in males [32], but no effect was observed in females [30, 32]. Strength-training had no association with early adult PA in males [32].

### Mental health

In total, seven studies considered mental health outcomes; six investigating depressive symptoms [35–39, 41], and three studying anxiety symptoms [38, 40, 41]. With a total of 16,592 observations, four studies utilised data from the Nicotine Dependence in Teens (NDIT) study [37–40], and two studies employed data from the National Longitudinal Study of Adolescent Health (Add Health) study [36, 41]; both North American cohorts. Ages ranged from 12 to 18 years at baseline and 20 to 32 years at outcome measurements. Mental health outcomes were assessed using self-report questionnaires and included depressive symptoms, suicidal ideation and attempts, generalised anxiety disorder, stress, coping, panic disorder, social anxiety, and agoraphobia. PA exposures in adolescence included team and individual sports, American football, and contact sports.

Adolescent team sport participation was associated with a reduced likelihood of early adulthood depression compared to no sport participation [39] and no organised team sport participation [37], however this association was minimised after accounting for confounders in one study [37]. American football participation was significantly linked to lower odds of depression in early adulthood in one [36] of two studies [36, 41] when compared to no football participation. No association was found for contact sports [36] or individual sports participation [35, 39].

No association was observed between adolescent team or individual sports participation and generalised anxiety disorder in early adulthood [38, 40, 41]. Despite this, one study found reduced odds of poor coping and stress in those that sustained team sports compared to no participation or discontinuation [38]. Furthermore, both team sports [38, 40] and individual sports [40] participation reduced the odds of panic disorder in early adulthood with a similar magnitude of effect. Participation in individual sports in adolescence was associated with lower odds of social anxiety in early adulthood [40], whereas team sports showed no effect [38, 40]. Adolescent team sports participation was associated with reduced odds of early adulthood agoraphobia in both studies that examined this relationship, when compared to no team sports participation [38, 40].

### Cardiovascular health

Three studies examined the association between adolescent PA types and cardiovascular health outcomes in adulthood, totalling 6,173 observations [33, 34, 41]. The outcomes measured included BMI, waist circumference, skinfolds, changes in overweight status from adolescence to adulthood, and diagnosis of high cholesterol, high triglycerides, hypertension, and heart disease. Two studies used data from the United States study, Add Health [34, 41], and one from the NDIT cohort based in Canada [33]. Ages ranged from 12 to 18 years at baseline and 18 to 32 years at follow-up.

Running in adolescence was associated with reduced adult waist circumference, BMI, and skinfold measures in early adulthood, however this association was no longer present after adjusting for adolescent body composition [33]. After adjusting for baseline body composition, sports participation (i.e., ice hockey, ice skating, football, roller blading/skateboarding, basketball, soccer, boxing and baseball) was positively associated with BMI in early adulthood [33]. Conversely, sports participation of specifically 3–4 days per week during adolescence was associated with lower odds of being overweight in early adulthood [34]. One study observed a potential association of dance and fitness participation and higher adulthood skinfold measures [33], however another study found no association [34]. Engaging in rollerblading, skating or cycling at least four days per week was associated with lower odds of being overweight or becoming overweight in early adulthood [34].

## Discussion

This was the first study to systematically review published research investigating the relationships between participation in specific types of LTPA in childhood and adolescence and PA outcomes and health in adulthood. Fourteen relevant studies from five countries were identified. Findings from this review suggest there are varying associations of adolescent participation in different leisure-time physical activities on long-term health and behavioural outcomes. Additionally, some evidence suggests these associations may be observed only after a certain dosage of participation, dependent on the type of activity. Specifically, exploring adulthood PA outcomes, adolescent running participation was consistently associated with higher early to mid-adulthood PA levels in both sexes [28–30, 32], as was sports participation in males [30–32]. One study showed that any number of years of running participation was associated with these benefits, while a minimum of four years of participation was required to observe such findings for sports participation [29]. Considering mental health outcomes, team sports were associated with a reduced risk of depression, panic disorder and agoraphobia [37–40], while individual sports were associated with a lower risk of social anxiety and panic disorder [38, 40]. Studies investigating cardiovascular outcomes were limited, with varying results making associations unclear.

### Physical activity

For the majority of LTPA types, participation in adolescence promoted continuation or re-uptake of the same activity in early adulthood [30]. This is consistent with the knowledge that perceived sport competence contributes to continual PA participation [42]. However, this association does not necessarily translate to higher total PA or LTPA levels in adulthood. Several studies observed varying effects of LTPA participation type in adolescence, and total PA in early to mid-adulthood, though these observations differed based on sex and study design [28–32].

Adolescent participation in running and running-based activities, including track and field and orienteering, were consistently correlated with higher overall PA levels in early to mid-adulthood [28–30, 32]. Even one year of participation in running-based activities during adolescence increased the likelihood of higher future PA levels [29]. Running is a relatively low-cost activity, requiring minimal resources and skills to undertake. Furthermore, it forms the basis of many other leisure-time activities, such as sports. These characteristics may allow adolescents to form a positive association with PA which is translated into adulthood behaviours.

Walking and hiking at 13 years of age was also associated with greater overall PA at age 23 years in both sexes [30], however these findings were not consistent with studies that measured PA outcomes at age 31 [32] and age 53 [28]. These findings suggest that walking in adolescence may positively impact PA during the transition to early adulthood, but potentially not at later ages. This period of early adulthood can signify a major transition period where many individuals are entering the workforce, moving out of home, and having children. As such, PA behaviours are susceptible to change at this time and adolescent PA associations may not be observed long-term [43].

Adolescent sports participation was positively associated with future PA in men, but not women [30–32]. Bélanger and Sabiston, et al. [29] identified that a minimum duration of four years sports participation during adolescence was required to observe such benefits, supporting the requirement for consistent participation during these formative years. Such sporting activities are often promoted in educational and recreational settings during the early years [15]. However, while these activities may promote lifelong PA in adolescent boys, these same effects may not be applicable to females. With females meeting fewer activity requirements than their male counterparts globally [9], it is particularly important to identify activities that will encourage lifelong PA in females, and that these activities are implemented in public health policy.

Dance and gymnastics-based activities had a positive association with future PA in only one of three studies when considered collectively [28, 29, 32]. Separately, gymnastics had a positive association in women [32]. While two studies showed no association of swimming in adolescence on later PA [44], another found a negative effect on overall PA at age 23 in females [30]. This observation warrants further research to determine the underlying motivations and experiences behind such changes during the transition to adulthood, particularly within a gendered context.

No studies examined the impact of LTPA types on objectively measured PA, with all five PA-based studies utilising self-report questionnaires. Although required for the classification of PA types, self-reporting methods can introduce misclassification and bias due to recall errors and social desirability [45–47]. Therefore, future research should focus on adopting a combination of objective and self-report PA measures where possible [48].

### Mental health

Studies investigating mental health outcomes considered only the exposures of team sports, individual sports, American football, and contact sports [35–41]. Participating in team, but not individual, sports during adolescence was associated with a reduced risk of early adulthood depression [37, 39]. All studies considered baseline depressive symptoms as a confounder, therefore suggesting an independent effect of team sports participation. Due to the additional social benefits of team sports participation, such activities may provide more psychological benefits [49].

No associations were found between adolescent PA types and generalised anxiety disorder in early adulthood [38, 40, 41]. Both team and individual sports participation in adolescence, however, were found to reduce the odds of panic disorder in early adulthood [38, 40]. Compared to those who quit, sustained team sports participation was associated with reduced levels of poor coping and stress, emphasising the importance of continued participation [38]. Of note, individuals who dropped out or had never participated in team sports had similar mental health outcomes to those who never participated in team sports [38]. Generally, team sport involvement enhances social cohesion and connectedness, minimising social isolation, and subsequently improving mental health [38]. This observation may potentially arise from the loss of social identity that accompanies ceasing team sport participation during the transition to adulthood [38].

Team sports participation was also associated with reduced odds of early adulthood agoraphobia [38, 40]. Given agoraphobia is characterised by an anxiety that occurs in crowds and public places, and the exposure to these environments is often unavoidable in team sports, this may desensitise individuals to such environments [40]. However, one study did not consider baseline agoraphobia or mental health symptoms as a covariate [40]; therefore, this association may be attributable to pre-existing symptoms hindering initial sports participation. Contrarily, participating in individual sports during adolescence was associated with reduced odds of social anxiety in adulthood [40], but this was not observed for team sports [38, 40].

### Cardiovascular health

Few studies examined the influence of specific PA types in childhood and adolescence on cardiovascular health outcomes and risk factors in adulthood [33, 34, 41]. While an association between running in adolescence and improved adult body composition was observed, this was no longer evident after considering baseline body composition [33]. No clear association was found for participation in dance activities [33, 34]. Menschik and Ahmed, et al. [34] reported reduced odds of being overweight based on adolescent participation in skating, rollerblading and cycling activities a minimum of four times per week [34].

Sports participation in adolescence was associated with higher adulthood BMI in one study [33]. This unexpected finding may potentially be attributable to the greater muscle mass generally observed in athletes, as these findings were not consistent in the skinfold or waist circumference measurements [33]. Conversely, another study found that participating in sports 3–4 days per week in adolescence was associated with lower odds of being overweight in adulthood [34]. These differences may also be reflective of the differences in study length and frequency of follow-up, with one study considering the number of years of participation over a five-year period [33], and another assessing only a single seven-day period at baseline [34]. This may further affirm the potential of a minimum level of adolescent sports participation before cardiovascular health benefits are observed in adulthood.

### Strengths and limitations

The strengths of this study include the rigorous systematic review approach as reported in line with the PRISMA statement [25]. The scope of the review was clearly predefined with explicit inclusion and exclusion criteria. A wide number of databases were searched for eligible studies, with several test searches conducted to strengthen the likelihood of capturing all relevant studies in the review. Study screening and data extraction were performed independently by two researchers, which has shown to be more reliable than single screening processes [50]. This systematic review was also strengthened by the inclusion of only prospective longitudinal studies. Furthermore, seven of fourteen studies included in the review were of good quality, with none rated as poor.

Studies were eligible for inclusion in the review if they were peer-reviewed and published in English; therefore, relevant grey literature and non-English studies may have been excluded. Though search terms were rigorously defined, it is possible that these terms did not encapsulate all potentially eligible studies. Due to between-study heterogeneity in exposure and outcome measures, a meta-analysis was not feasible in this review, subsequently limiting the ability to present quantitative evidence.

While the inclusion of only prospective longitudinal studies is a strength, limitations may arise from the presence of selection bias resulting from inevitable loss to follow-up. Additionally, baseline periods of the included cohorts ranged from 1968 to 2001; therefore, findings may differ to present-day observations. For example, in Australia, there has been an overall increase in female sport participation, mostly led by male-dominated sports such as soccer, Australian rules football and cricket [51]. With increasing availability of resources and professional opportunities for women in sport, there may have been activities excluded from current studies that have lifelong benefits for women.

### Knowledge gaps and future directions

Numerous gaps in the evidence-base were identified. Firstly, there were no studies that considered PA in childhood, with all included studies commencing baseline no earlier than 12 years of age. This may be of particular importance, as early sport commencement has been found to improve retention [52]. Secondly, research was geographically limited to five countries (Canada, United States, Sweden, Norway, and Finland), so findings may not be applicable to other regions. As different countries are known to participate in different activities due to variances in climate, culture, and resources [15], research in other countries is required to better inform how region-specific resourcing is best directed. Thirdly, few studies considered exposure activities separately [28, 30, 32], with many grouping activities prior to analysis using either logic-informed [28, 31, 34] or statistical methods [29, 33]. Overall, there were a limited number of studies that considered different leisure-time activity types and their different associations with long-term health and behavioural outcomes.

A particularly limiting factor to results synthesis was the variation in the definition and categorisation of sport and LTPA types, which has also been identified in previous reviews [22]. As such, developing a universal method to categorise activities would be of benefit for future research. Exploring the feasibility of utilising data-driven grouping methods alongside logic-informed categorisations could contribute to the development of universally accepted approaches to grouping LTPA types. Exploratory factor analysis has previously been used to categorise activities based on data from the NDIT study, however this approach resulted in excluding a significant number of activity types [29]. Research should continue to develop methods that are inclusive of more activities.

Previously, government funding in many countries has been largely directed towards developing elite sporting programs and athletes [53]. A recent shift in prioritisation to health-enhancing and ‘Sport for All’ approaches in many countries has allowed for the redirection of funds and resources to focus on lifelong PA participation [53, 54]. As trends shift globally, the expansion of knowledge and education of the longitudinal benefits of PA types will contribute to creating active societies, environments, people, and systems; pillars identified by The World Health Organisation 2018–2030 Global Action Plan on Physical Activity [18]. Such knowledge will contribute to the implementation of best practice communication campaigns and education, inform mass participation initiatives, direct resources and funding, and strengthen policy frameworks to ultimately promote lifelong activity and good health.

## Conclusions

This review identified fourteen studies that investigated associations between adolescent participation in specific types of LTPA and PA, mental health, and cardiovascular health outcomes in adulthood. No studies that considered childhood PA participation were identified. Findings suggest preliminary evidence that the lifelong effects of adolescent LTPA participation may be dependent on the type of activity, with potential differences between sexes. Furthermore, there is some evidence to suggest that minimum thresholds of participation for certain activities may be required before future behaviour and health benefits are observed. Most notably, running throughout adolescence was associated with more favourable physical activity outcomes. Adolescent sports participation in men showed associations with higher adulthood physical activity. In both men and women, team sports participation was associated with lower levels of adulthood depression and agoraphobia. Further research is required to confirm the preliminary evidence identified in this review, with the inclusion of other physical activity types and health outcomes to accurately inform policy and practice. Future studies in a range of countries should be conducted to inform local policymakers and communities on where funding and resources may be most beneficially distributed to effectively promote lifelong health and PA participation.

### Electronic supplementary material

Below is the link to the electronic supplementary material.

Additional file 1: PRISMA 2020 checklist Completed PRISMA 2020 checklist for reporting on systematic reviews, including page numbers and table/figure numbers referencing where the guideline has been addressed.

Additional file 2: Full search strategy by database Full search strategy for each of the five databases used during the systematic search.

Additional file 3: Modified Newcastle-Ottawa quality assessment form for cohort studies List of all criteria for risk of bias assessment as per the modified Newcastle-Ottawa scale, including rating criteria.

## Data Availability

The data analysed during this study are available from the corresponding author upon reasonable request.

## Abbreviations

Add Health: National Longitudinal Study of Adolescent Health
BMI: Body mass index
LTPA: Leisure-time physical activity
NDIT: Nicotine Dependence in Teens
NOS: Newcastle-Ottawa Scale
PA: Physical activity
PRISMA: Preferred Reporting Items for Systematic Reviews and Meta-Analyses
