# Supplementary material for: Types of leisure-time physical activity participation in childhood and adolescence, and physical activity behaviours and health outcomes in adulthood: a systematic review
Source: BMC Public Health. 2024 Jul 4;24:1789. doi: 10.1186/s12889-024-19050-3 (PMC11225122; doi:10.1186/s12889-024-19050-3)
Supplement: Supplementary file 1 — Additional file 1: PRISMA 2020 checklist Completed PRISMA 2020 checklist for reporting on systematic reviews, including page numbers and table/figure numbers referencing where the guideline has been addressed. [file 12889_2024_19050_MOESM1_ESM.docx]

##### ADDITIONAL FILE 3

###### Modified Newcastle-Ottawa quality assessment form for cohort studies

**SELECTION 1: Representativeness of the sample**

1. **Truly** representative of the source population – randomly selected or all participants invited ***(two stars)***
2. **Somewhat** representative of the source population – non-random selection ***(one star)***
3. **No description** of the derivation of the cohort

**SELECTION 2: Ascertainment of childhood/adolescent physical activity participation**

1. **Self-report** during childhood/adolescence by participants/parents ***(one star)***
2. **Secure record** (e.g., school records) ***(one star)***
3. **Retrospective** collection in adulthood
4. **No description**

**COMPARABILITY 1: Comparability of cohorts on the basis of the design or analysis controlled for confounders**

1. **Comparable** - the study controls for relevant factors (e.g., age, sex) ***(one star)***
2. **Not comparable** - relevant factors are not controlled for

**OUTCOME 1: Assessment of outcome**

1. **Objective** methods ***(two stars)***
2. **Record linkage** ***(two stars)***
3. **Self-report** ***(one star)***
4. **No description**

**OUTCOME 2: Adequacy of cohort follow-up**

1. **Complete** follow up - all subjects accounted for ***(one star)***
2. **Acceptable** - subjects lost to follow up unlikely to introduce bias. Number lost is less than or equal to 20% or description of those lost suggested no different from those followed ***(one star)***
3. **Insufficient** - follow up rate less than 80% and no description of those lost
4. **No statement**

Maximum score = 7

**Good quality** ≥ 6 stars

**Fair quality** = 4 or 5 stars

**Poor quality** ≤ 3 stars

| **Study ID** | **Quality rating** | **Modified Newcastle-Ottawa Quality Assessment Scale** | **SELECTION 1: Representativeness of sample** | **SELECTION 2: Ascertainment of exposure** | **COMPARABILITY 1: Comparability** | **OUTCOME 1: Assessment of outcome** | **OUTCOME 2: Adequacy of follow-up** |
| --- | --- | --- | --- | --- | --- | --- | --- |
| Ashdown-Franks, 2017 | Fair | 5* | Truly** | Self-report* | Comparable* | Self-report* | Insufficient |
| Belanger, 2015 | Good | 6* | Truly** | Self-report* | Comparable* | Self-report* | Acceptable* |
| Belanger, 2018 | Good | 6* | Truly** | Self-report* | Comparable* | Objective** | Insufficient |
| Bohr, 2019 | Fair | 5* | Truly** | Self-report* | Comparable* | Self-report* | Insufficient |
| Brunet, 2013 | Good | 6* | Truly** | Self-report* | Comparable* | Self-report* | Acceptable* |
| Deshpande, 2020 | Good | 6* | Truly** | Self-report* | Comparable* | Self-report* | Acceptable* |
| Engström, 2008 | Fair | 5* | Truly** | Self-report* | Not comparable | Self-report* | Acceptable* |
| Kjonniksen, 2008 | Fair | 5* | Truly** | Self-report* | Not comparable | Self-report* | Acceptable* |
| Mäkelä, 2017 | Fair | 5* | Truly** | Self-report* | Comparable* | Self-report* | Insufficient |
| Menschik, 2008 | Fair | 5* | Truly** | Self-report* | Comparable* | Self-report* | Insufficient |
| Murray, 2021 | Good | 6* | Truly** | Self-report* | Comparable* | Self-report* | Acceptable* |
| Sabiston, 2016 | Good | 6* | Truly** | Self-report* | Comparable* | Self-report* | Acceptable* |
| Tammelin, 2003 | Good | 6* | Truly** | Self-report* | Comparable* | Self-report* | Acceptable* |
| Viau, 2015 | Fair | 4* | No description | Self-report* | Comparable* | Self-report* | Acceptable* |

**Appendix D Table 1.** Quality assessment table for studies included in the systematic review, based on a Modified Newcastle-Ottawa Scale.
