# Supplementary material for: Types of leisure-time physical activity participation in childhood and adolescence, and physical activity behaviours and health outcomes in adulthood: a systematic review
Source: BMC Public Health. 2024 Jul 4;24:1789. doi: 10.1186/s12889-024-19050-3 (PMC11225122; doi:10.1186/s12889-024-19050-3)
Supplement: Supplementary file 2 — Additional file 2: Full search strategy by database Full search strategy for each of the five databases used during the systematic search. [file 12889_2024_19050_MOESM2_ESM.docx]

**ADDITIONAL FILE 2**

**Full Search Strategy by Database**

EMBASE via Ovid

*559 results*

(((sport* or physical activit* or exercise) adj5 (youth or childhood or adolescen* or school or teen*)) and (adult or adulthood or midlife) and (type* or subtype* or participation)).ab,kf,ti.

Medline via Ovid

*422 results*

(((sport* or physical activit* or exercise) adj5 (youth or childhood or adolescen* or school or teen*)) and (adult or adulthood or midlife) and (type* or subtype* or participation)).ab,kf,ti.

Scopus

*1,923 results*

( TITLE-ABS-KEY ( ( youth OR childhood OR adolescen* OR school OR teen* ) W/5 ( sport* OR "physical activit*" OR exercise ) ) ) AND ( TITLE-ABS-KEY ( type* OR subtype* OR participation ) ) AND ( TITLE-ABS-KEY ( adult OR adulthood OR midlife ) )

Web of Science

*403 results*
(TI=((sport* OR "physical activit*" OR exercise) NEAR/5 (youth OR childhood OR adolescen* OR school OR teen*))) OR (AB=((sport* OR "physical activit*" OR exercise) NEAR/5 (youth OR childhood OR adolescen* OR school OR teen*))) OR (KP=((sport* OR "physical activit*" OR exercise) NEAR/5 (youth OR childhood OR adolescen* OR school OR teen*))) AND ((TI=(type* OR subtype* OR participation)) OR KP=(type* OR subtype* OR participation)) AND ((AB=(type* OR subtype* OR participation)) OR ((TI=(adult OR adulthood OR midlife)) OR KP=(adult OR adulthood OR midlife)) OR AB=(adult OR adulthood OR midlife)))

SPORT Discus

*459 results*

((TI ( sport* OR "physical activit*" OR exercise ) OR KW ( sport* OR "physical activit*" OR exercise ) OR AB ( sport* OR "physical activit*" OR exercise )) N5 (TI ( youth OR childhood OR adolescen* OR school OR teen* ) OR KW ( youth OR childhood OR adolescen* OR school OR teen* ) OR AB ( youth OR childhood OR adolescen* OR school OR teen* ))) AND (TI ( type* OR subtype* OR participation ) OR KW ( type* OR subtype* OR participation ) OR AB ( type* OR subtype* OR participation )) AND (TI ( adult OR adulthood OR midlife ) OR KW ( adult OR adulthood OR midlife ) OR AB ( adult OR adulthood OR midlife ))
